# Students’ experiences of learning in relation to didactic strategies during the first year of a nursing programme: a qualitative study

**DOI:** 10.1186/s12909-015-0338-x

**Published:** 2015-03-17

**Authors:** Lars Westin, Annelie J Sundler, Mia Berglund

**Affiliations:** 1 School of Health and Education, University of Skövde, Skövde, Sweden; 2 School of Health, Care and Social Welfare, Mälardalens University, Västerås, Sweden

**Keywords:** Nursing, Education, Teaching, Qualitative, Learning strategies

## Abstract

**Background:**

In university undergraduate nursing programmes, didactic strategies that enable students to learn nursing skills, solve problems and develop reflective and critical thinking and practice are needed. The aim of this study was to explore how different didactic strategies support nursing students’ experiences of learning during the first year of a reconstructed nursing curriculum.

**Methods:**

This study employed a qualitative approach. The data were gathered through written narratives that were analysed using qualitative content analysis.

**Results:**

Nursing students’ experiences of learning through different didactic strategies, were evident in the text. These perspectives were organised into the following themes: To focus on the patient perspective and paying more attention to others, Learning from discussions and reflections on one’s own learning, Training for the professional role and becoming more courage, and Gaining insights into nursing and increasing one’s self-awareness. The education increased the students’ self-awareness, which helped them to pay greater attention to patients and their relative. During the learning process, the students became more courageous, reflected and discovered their shortcomings.

**Conclusion:**

Stated didactic strategies supported a broad base of knowledge on nursing and the professional role of nurses. Educators are challenged to strengthen meaningful learning in nursing and to facilitate the progression of nursing programmes.

## Background

The goal of undergraduate nursing education is to provide opportunities for students to become nurses with the knowledge and skills that are needed to provide high-quality care based on patients’ needs. According to Ramsden [1], teaching in higher education does not solely concern students’ ability to recall or reproduce information. Rather, it concerns altering students’ understanding, which involves a qualitative change in the students’ view of reality. Strategies that enable students to develop nursing skills, solve problems and develop reflective and critical thinking are needed in university undergraduate nursing programmes. Ramsden [1] highlights the importance of students’ actions while learning and states that good teaching provides students with the opportunity to learn. From this point of view, knowledge about the aspects that impact nursing students’ learning is needed. Therefore, the current study was conducted to develop knowledge about how stated didactic strategies support nursing students’ learning during the first year of a reconstructed nursing programme.

Reflective and critical thinking is important in higher education in general and in undergraduate nursing education in particular [2-4]. In the field of nursing, learning has shifted from ‘doing’ to ‘understanding’ [5]. Currently, health care professionals who are self-directed, autonomous and able to think critically are needed. This need may partially explain why problem-based learning (PBL) has become widespread in nursing education [6]. Levett-Jones [7] highlights that a self-directed approach to learning (SDL) can increase nursing students’ confidence in their ability and their capacity to learn in new situations. The SDL approach has also received attention in nursing education because it is human-oriented and can be associated with professional autonomy. In preparing a professional nurse for practice, lifelong learning must be emphasised. Levett-Jones states that, in a constantly changing environment, SDL can be an essential vehicle that enables students to develop independent learning skills. Regan [8] notes teachers’ important role in motivating and inspiring students to learn and calls for an open perspective concerning the range of factors that motivate students’ learning.

The integration of theory and practice has been identified to be a challenge in nursing education [4,9-11]. Due to these challenges, students may find it difficult to understand theoretical knowledge and the practicality of that knowledge. Both theory and practice are essential, and several studies have highlighted the importance of clinical practice in nursing programmes [12,13]. In research, reflection has been pointed out as central for the linking of theory with practice in the learning process [14-17]. Ekeberg [4] argues that a lifeword perspective on both learning and caring is important to enable this linking.

Applying a lifeworld approach to learning [4,18,19] enfolds a holistic approach, which means that thoughts, feelings, theoretical and practical experiences and embodied understanding are involved in the learning process. According to the theory of the lifeworld we usually have a natural attitude in everyday life, witch basically is unreflective [18]. However, our natural attitude and experiences can be examined and conceptualised through reflection. Thus, experiences can be made aware and available for analysis instead of merely being taken for granted [4]. Through reflection, the student can work with meanings, value sets and approaches. This makes discovering and reconsidering of new versions of experiences and attitudes possible [20]. Consequently, the lifeworld can be seen as a platform for learning. From that point of view learning needs to be understood in relation to the individual and his or her experiences, which are related to his or her learning.

The theory of the lifeworld has been described by the philosopher Husserl [18] and further developed by Merleau-Ponty [19]. From a lifeworld perspective the learning process for nurse students can be understood as an encounter between the student’s lifeworld, scientific knowledge and best practice (4). Didactic strategies are needed in order to provide support and create meaningful conditions necessary for a reflective process that strengthens the integration between the student’s lifeworld and theoretical and practical knowledge. In undergraduate nursing education, such reflection involves a process of understanding of theoretical knowledge in relation to clinical nursing practice. Yet, such reflections are needed on experiences from both theoretical and clinical education. Research that focuses on didactic strategies to support learning in this area is lacking.

At the local university where this study was conducted, the three-year nursing programme curriculum with a Bachelor’s degree was reconstructed. The new curriculum was based on comprehensive work focused on quality and development. One aspect of this work was to develop the nursing discipline in the program. A second aspect was to develop didactic strategies for teachers that would harmonise with the main focus of the nursing programme. The university’s main concern was to build a curriculum that was based on patient-centeredness [21,22] supported by three didactic strategies. The main focus in the curriculum is Patient- centeredness based on a humanising care approach to nursing, in which the patient’s perspective and experiences are considered. Both the approach and the didactic strategies that were developed were based on the lifeworld theory [18,19]. Ekeberg [4] highlights that didactic strategies must be based on the same theoretical basis as the main subject in the education curriculum. The first didactic strategy is Patient-centeredness, the second strategy is to intertwine theory and practice and the third strategy is that teaching should facilitate the development of an ethically reflective approach. With these concerns in mind, the nursing curriculum was reconstructed. In each semester, courses that included both theoretical and clinical aspects were offered. Furthermore, theory was combined with clinical education at either the Clinical Training Centre (CTC) or during Clinical Placements (CP). The first year of the programme included courses on professional nursing, values and core concepts, communication skills, nursing ethics, public health, anatomy, physiology, microbiology and hygiene.

The reconstructed nursing curriculum and the didactic strategies were evaluated after the first year. To verify the value of such teaching strategies, knowledge about the students’ learning processes is needed. In addition, studies that focus on facilitating and supporting students’ learning and understanding of nursing are needed. Therefore, in the current study, we invited first-year students to describe their learning experiences. The aim of this study was to explore how different didactic strategies support nursing students’ experiences of learning during the first year of a reconstructed nursing curriculum.

## Methods

The current study utilised a qualitative approach. The data were gathered through written narratives that were analysed using qualitative content analysis, [23-25]. Qualitative content analysis focuses on the analysis of written texts, particularly within areas such as human sciences and health sciences. The overall objective of content analysis is to describe the similarities and differences in the content of the text. Specifically, the goal is to describe the central content of the text. These similarities and differences are then expressed in different themes. In this study a manifest content analysis was conducted, such an analysis refers to what the text says and describes the visible and obvious content of the text rather than the underlying meaning [23]. In the current study, nursing students were asked to write a report in which they expressed their experiences of learning. The students were asked questions about their experiences of their learning during the first year of their education and what they perceived as meaningful during this learning.

### Selection of participants

All students in the nursing programme were informed of the study both orally and in written text. Written consent to participate in the study was obtained from the students. The head of the department also approved the current study. All of the students (N = 38) participated in the study. The students were aged between 49 and 21 years (M = 27). Of these 25 were women and 13 men.

### Data collection

The data were gathered from students on two occasions, at the end of the first semester and at the end of the second semester. At the end of the first semester, the students were asked to submit a written narrative outgoing from three questions in which they described with their own words how they have learned in different educational situations trough their learning process. The questions concerned their 1) learning experiences; 2) what was important for their learning; and 3) experiences of different didactic strategies that has support their learning. At the end of the second semester, the students were again asked to submit another written narrative in which they described how they have learned in their coursework to date, based on the same questions. The students were also asked about their views on the nursing profession and if their thinking about their future professional role had changed. The collected data set consisted of approximately 125 A4 pages of written text. The length of the narratives varied from one to three A4 pages.

### Data analysis

Content analysis was employed [23-25]. The analysis was performed as follows. First, two of the authors read through all the text from both occasions several times to obtain a first impression of the content and the topic of the text. Second, various meaning units from the text were selected. Third, the meaning units were condensed into shorter and stronger core descriptions of the text content. Fourth, the condensed text was evaluated for similarities and differences, resulting in distinctive themes. The third author continuously read and gave comments on the analysis, to ensure that the validity of the description and that the themes covered the data. The analysis process is illustrated with examples in Table 1.

**Table 1:** **An example of the analysis process**

Meaning units	Condensed text	Themes
Has gained greater insight from the patients’ perspective to better understand and involve the patients Has developed and increased an awareness of nurturing the whole patient	An increased understanding of the patients’ perspective makes it easier to involve patients. The awareness of caring for the whole patient has increased	Focus on the patient perspective and paying more attention to others
Working in study groups motivates one to engage more in studies and provides greater learning	It is motivational and educational to work in study groups	Learning from discussions and reflections on one’s own learning

### Ethical aspects

All students received written and verbal information about the study’s purpose. The information clearly stated that participation was voluntary and that an unwillingness to participate would have no consequences on their education. The information also noted their right to withdraw from the study and that all data were confidential (i.e., a participating student cannot be identified). The study was performed in accordance with the Code of Ethics of the World Medical Association (Declaration of Helsinki) and according to Swedish National Board of Health and Welfare (SFS 2003:460).

## Results

Nursing students’ experiences of learning through different didactic strategies, were evident in the text. These perspectives were organised into the following themes: 1. To focus on the patient perspective and paying more attention to others, 2. Learning from discussions and reflections on one’s own learning, 3. Training for the professional role and becoming more courage, 4. Gaining insights into nursing and increasing one’s self-awareness. These themes are presented below, with selected quotes.

### To focus on the patient perspective and paying more attention to others

One important aspect of learning was based on the patient’s perspective. This goal could be achieved because the students were given the opportunity to reflect on the patient’s perspective in seminars and by reading patients’ stories. The students stated that they had to challenge themselves to understand what the patient perceives as important in care. This perspective was expressed in lectures, seminars and study assignments.

*“The understanding of the patient’s perspective can never be obtained as a caregiver without first asking what the patient thinks is important. The things the nurse considers to be small things can be huge for the patient.”*

Students stated that watching movies that involved patient scenes impacted this aspect of their learning. Through this method, the students developed a new understanding of patients’ experiences in relation to the students’ professional role. The movies that were shown in the course helped deepen the students’ understanding of the patient’s perspective and provoked their own reflections. During the courses, students were advised to maintain diaries to facilitate their reflections. The format and the amount that was recorded in the students’ diaries differed. Some students maintained a diary from the beginning of the course but later experienced difficulties in continuing these reflections. Furthermore, the students were trained to reflect on the patient’s perspective and the nursing role during practical training at the CTC and during CP.

*“As to the patient’s perspective, I think it has markedly evolved in me during the courses. I think it’s important to remember that every conversation with a patient is unique and that you cannot take for granted how a patient will respond or how a patient perceives what you say to him - for all perceive things differently.”*

Students reported an improved ability to listen in conversations with others. They also became more open and attentive in communicating with other people. This skill was represented as an increased sensitivity and awareness of how their communication with patients impacted care. The students also expressed the importance of showing interest and engagement in the patients’ stories.

*“I’ve become more sensitive after these courses. I listen more to both my wife and friends. I have also become better at seeing what is not said.”*

*“I understand that it is often the small things that can make the most of a person. Small things for me could be that I sit down and talk with the patient. To me, it’s no sacrifice at all; I like to sit down and talk and listen to what they have to tell. But for the patient, it may mean a lot more than I can understand.”*

Students described that they became increasingly aware that patients perceive health professionals’ signals and attitudes and that the students’ approach was important. The students stated the importance of understanding their personal experiences and feelings such that these factors would not hinder the care of the patient.

### Learning from discussions and reflections on one’s own learning

Discussion within study groups was also reported as an aspect of learning. All of the students were divided into small groups of four to five students. These students collaborated on various learning tasks that were reported in follow-up seminars. The students expressed that they learned from each other in these groups.

*“Our study group has been a tremendous support for us; the study group motivates us to become more engaged in our studies.”*

The students also highlighted that seminars were important. Students expressed that they greatly learned from each other’s views during discussions and seminars. These experiences demonstrated the students’ different perceptions of problems.

In the written response, the students expressed that they had opportunities to develop as a caregiver during the first year of the nursing programme. The students stressed that they matured both as a person and as a caregiver. They also stated that they gained an increased awareness of caring for the patient through a new view of the world, the patient and reality. In reflecting on their own learning, the students reported that their first year in the nursing programme led to personal development. Such development and improved self-confidence motivated the students to study and learn more.

*“I carry out different things, dare to grab things - self-confidence grows from this.”*

After the first year in the nursing programme, the students expressed that they had begun the journey of becoming a nurse. Although the students had much more to learn before becoming nurses, their knowledge increased and they experienced growth through their learning.

### Training for the professional role and becoming more courage

The students reported that the nursing skills training in CTC prior to their clinical placements was important. This training helped prepare them for the care that they would provide in real situations. The students considered the practice and training of various skills to be both instructive and enjoyable. The students also enjoyed role-playing in CTC as a way of learning, especially when acting as patients, relatives or nurses during role-play.

*“CTC has been very instructive; it is one thing to read about how to do and then another thing to actually do it. CTC was interesting when we got to act as both the patient and the nurse. It has been useful to act as the patient. The patient must be able to show what she wants. It was important to reflect on this with the patients’ perspective in the CTC.”*

Even the students who had previous extensive experiences as assistant nurses stated that they learned a great deal during the CTC training. The students reported that they deepened their knowledge, improved their nursing skills and learned from each other during the training. Students experienced the vulnerable and disadvantaged role of the patients through role-play. The experiences of the inability to brush one’s teeth or to eat food at one’s preferred speed were important insights for the students.

The nursing students stated that they became more courageous in various situations. They trusted their ability and developed increased self-confidence. The students stated the importance of challenging their abilities and being themselves. The students expressed a need for confirmation; they wished to ensure that they were performing tasks correctly. This confirmation strengthened their confidence and improved their courage for subsequent tasks.

*“This course has made me understand that I know more than I think I do. It has made me dare to straighten my back and actually stand for what I think of something. I believe this is largely thanks to the various seminars.”*

### Gaining insights into nursing and increasing one’s self-awareness

The students reported that a clinical placement early in the first semester was valuable for their learning. They became involved in real patient situations and had opportunities to care for patients in the early stages of the programme; however, they stated that it was valuable to observe a different care situation through CP.

*“Being at a nursing home has been very enjoyable. I apply the lessons learned in school and observe how caregivers communicate with the residents. I also learn what not to do in different care situations.”*

Through a new theoretical understanding of caring (e.g., ethical theories and various communication strategies) and CP, the students developed a new understanding and more critically reflected on their nursing actions. Students with extensive health care experience noted problems with their earlier experiences that they did not previously consider. They discovered many ways to care for patients and that they needed to utilise new strategies and knowledge for each unique situation.

By practicing their knowledge in performing role-plays or practical training, the students could discover their own shortcomings. Through practice, they gained insight into how to perform and improve their nursing behaviours. For example, the students discovered shortcomings in their communication with patients and the significance of their body language in communication.

*“I think it’s good that you may wonder how one thinks and acts. Then you can figure out how you are and how you can change what is wrong.”*

The students highlighted that their studies in nursing increased their self-awareness through a deeper understanding of how to meet patients. Their knowledge and understanding of nursing created a strong foothold, and the students learned new things about themselves. This knowledge was used to better understand other people. They stated that this knowledge allowed them to show greater transparency when meeting people and to more easily accept the people as individuals.

*“I feel like I’ve evolved as a person and have learned to think differently. I feel that I can understand other people better now.”*

*“The clinical training has led to many insights, such as small things can bring more suffering and it is easy to offend people in different ways. The training has led to a greater understanding and sense of caring more about people.”*

## Discussion

The current study revealed that nursing students’ learning experiences during the first year of a nursing programme in Sweden involved developing an understanding of nursing knowledge and skills based on the patient’s perspective. Such knowledge and understanding were obtained through reading and discussing patients’ life stories and movies that illustrated patients’ lived experiences, which can be related to the first of the learning strategies. Hörberg and Ozolins [26] show that movies on caring for patients may enhance students’ understanding of caring and the patient’s care needs.

The findings indicated that students deepened their understanding through reflections and discussions, such as through small study groups. In the group discussions, the students learned from each other and reflected together. These discussions helped the students widen their perspectives. Working in small groups seemed to enhance the students’ critical and reflective thinking skills, which can be related to the third of the learning strategies. However, study groups can be negative for some students. Both advantages and disadvantages of work group processes have been described [27]. In Gagnon’s study [27], nursing students reported that their learning was enhanced by the diversity of ideas and viewpoints that were shared in work group processes. However, the willingness to learn from each other varied among students. Although some students welcomed feedback and discussions, other students were more reluctant in the sharing process. Therefore, teachers must carefully follow group discussions and continuously support study groups. To enhance learning in groups of nursing students, we argue that students should be provided with instructions and direction concerning the topics to learn and discuss.

Practical experiences in of different care situations also motivated students to learn. Through CP and CTC, the students developed impressions and views on their future professional role. They were also given the time to observe and learn from various care situations. The students described the combination of theory and practical training, such as role-playing in CTC, as meaningful and as a method of interweaving nursing theory into practical situations, which can be related to the second learning strategy. In learning to become nurses, learning from practice was meaningful for nursing students. Generally, nursing students report satisfaction with the clinical learning environment [28-30]. In the current study, the nursing students were satisfied with their clinical placements during their first year in the nursing programme. In other words, the clinical nursing situations that they experienced in these placements gave them new insights and a strong knowledge base.

In learning to become nurses, the students also described an increased self-awareness from their learning. This insight seemed to initiate a deeper focus on the patients’ perspective. Patients’ experiences and life stories are important in health care. Dahlberg et al. [21] emphasise that an existential view of well-being or illness in health care is needed and that patient experiences must be acknowledged. Patient experiences are important for the quality of care, and the patients’ views must be acknowledged to reduce and alleviate the patients’ suffering [31,32]. Accordingly, this approach should be introduced early in nursing education so that this knowledge is incorporated into the professional view of nursing. However, to progress and deepen this knowledge in a three-year curriculum may be challenging. The educational design of a nursing curriculum has been highlighted as important to students’ understanding of nursing [33]. In Fredholm-Nilsson and Silén’s study [33], problem-based learning increased the students’ awareness of the relation between education content and the nursing profession. Similar to that study, the current study found that the learning and development of professional nursing knowledge that began in the first year of nursing school supported the students’ reflections on their learning, thus increasing their awareness of their learning processes.

The students also expressed that they became more attentive and better listeners. This skill could be applied both in their personal lives and in clinical situations. The students more clearly understood the importance of listening to the patient. This skill was viewed both as a high form of moral sensitivity in which the patient was confirmed as an individual and as an improved understanding of the patient’s perspective. The didactic strategies from the examined nursing curriculum were theoretically grounded in lifeworld theory [4,18,19]. The study clearly showed that the students developed an initial understanding of this theory in their first year of school. This understanding also seemed to be integrated into the students’ clinical work. Future education should emphasise and deepen the core of this theory. The lifeworld theory focuses on and prioritises the patients’ perspective in care. A study by Park, Kjervik, Crandell and Oermann [34] showed that moral sensitivity was high in patient-oriented care.

Finally, the current results focused on students’ discovery of their shortcomings in clinical training and role-play, which increased their desire to continue developing and improving. This development increased their courage and ability to observe opportunities in each situation. The development of professional competence such that nurses are able to observe new possibilities and meaning of caring is crucial in a complex health care environment. The students in the current study stated that they became more courageous, trusted their ability and experienced increased self-confidence. This finding is consistent with Levett-Jones’ [7] claim that self-directed learning can increase nursing students’ confidence in their ability to learn from new situations. Finally, the students reported that they gained an increased awareness of caring for the patient through new views of the world, the patient and reality. In reflecting on their learning, the students stated that their learning during the first year in the nursing programme led to personal development.

### Strengths and limitations

The outcome of this study elucidates student nurses experiences of how different didactic strategies supported their learning during the first year of a reconstructed nursing curriculum. The study provides insights concerning the importance of didactic strategies based on the patient perspective to support nursing students’ learning and reflections in relation to both theoretical and practical knowledge. The strength of this study is that there is not much research done on this topic. However, the study was limited to a specific institution, and a restricted group of students, which may be a limitation of the study. Even though the students narratives were rich of meanings and descriptions, and the methodological approach used was found to be well suited.

## Conclusion

The findings revealed that the patient perspective was meaningful for the nursing students learning. Their learning was developed in student group discussions. They were trained in their professional role in the Clinical Training Centre and gained insights into nursing from clinical placements. Altogether, the stated didactic strategies supported a broad base of knowledge on nursing and the professional role of nurses.

Students’ learning was experienced as meaningful when they could develop their knowledge and gain new insights. Increased self-awareness, which helped them pay greater attention to patients and others, was important to the students. Meaningful learning experiences increased the students’ courage. Furthermore, the students were able to reflect and utilise critical and reflective thinking.

The findings indicate that the introduction to nursing education may influence student learning during the first year and probably also the following years of their education. This might even influence how they learn in their forthcoming professional roll as a nurse. The strategies that teachers and students utilise must be meaningful for the students. We challenge educators to strengthen meaningful learning that focuses on the patient perspective and facilitates the progression of nursing knowledge.

## Abbreviations

PBL: Problem-based learning
SDL: Self-directed approach to learning
CTC: Clinical training centre
CP: Clinical placements
